# Climate Response of Tree Radial Growth at Different Timescales in the Qinling Mountains

**DOI:** 10.1371/journal.pone.0160938

**Published:** 2016-08-10

**Authors:** Changfeng Sun, Yu Liu

**Affiliations:** 1 Department of Earth and Environmental Science, School of Human Settlements and Civil Engineering, Xi'an Jiaotong University, No.28, Xianning West Road, Xi'an, 710049, China; 2 The State Key Laboratory of Loess and Quaternary Geology, Institute of Earth Environment, Chinese Academy of Sciences, No.97 Yanxiang Road, Xi'an, 710061, China; Institute of Tibetan Plateau Research, CHINA

## Abstract

The analysis of the tree radial growth response to climate is crucial for dendroclimatological research. However, the response relationships between tree-ring indices and climatic factors at different timescales are not yet clear. In this study, the tree-ring width of Huashan pine (*Pinus armandii*) from Huashan in the Qinling Mountains, north-central China, was used to explore the response differences of tree growth to climatic factors at daily, pentad (5 days), dekad (10 days) and monthly timescales. Correlation function and linear regression analysis were applied in this paper. The tree-ring width showed a more sensitive response to daily and pentad climatic factors. With the timescale decreasing, the absolute value of the maximum correlation coefficient between the tree-ring data and precipitation increases as well as temperature (mean, minimum and maximum temperature). Compared to the other three timescales, pentad was more suitable for analysing the response of tree growth to climate. Relative to the monthly climate data, the association between the tree-ring data and the pentad climate data was more remarkable and accurate, and the reconstruction function based on the pentad climate was also more reliable and stable. We found that the major climatic factor limiting Huashan pine growth was the precipitation of pentads 20–35 (from April 6 to June 24) rather than the well-known April–June precipitation. The pentad was also proved to be a better timescale for analysing the climate and tree growth in the western and eastern Qinling Mountains. The formation of the earlywood density of Chinese pine (*Pinus tabulaeformis*) from Shimenshan in western Qinling was mainly affected by the maximum temperature of pentads 28–32 (from May 16 to June 9). The maximum temperature of pentads 28–33 (from May 16 to June 14) was the major factor affecting the ring width of Chinese pine from Shirenshan in eastern Qinling.

## Introduction

Climate studies are very important for obtaining the information on natural and human environments, and the paleoclimate is essential for the overall understanding of climate change. Due to their wide spread, precise date, high resolution, and high sensitivity to the climate, tree rings have been widely used in past climate reconstructions across many regions [1–7]. In dendroclimatological research, monthly climatic factors, such as monthly total precipitation and mean temperature, are consistently used to study the response relationship between tree-ring indices and climate. Under such circumstances, the continuous growth process of trees is mechanically separated, during which some of the climate signal from the tree ring is likely lost. If the tree response to climate is conducted at an appropriate timescale that corresponds to tree growth regularity, more accurate climate signals can be obtained from tree rings.

The living environment and climate factors, such as precipitation, temperature and soil moisture, are critical for tree growth [8, 9]. Because of the diurnal variation in climatic elements and tree physiological properties, the radial growth of trees exhibits small diurnal changes [10, 11]. Studies of tree growth mechanisms usually utilize daily climatic factors [12, 13]. Daily climatic factors play an important role in the formation of tree-ring width and density, especially the daily temperature in the pre- and early growing season [14]. In dendroclimatology, Feng et al. [15] noted that tree radial growth was more sensitive to daily temperature than to monthly temperature, and tree-ring series could be used to reconstruct daily cumulative temperature [16]. In addition to daily and monthly timescales, other common scales, such as pentad (5 days), weekly and dekad (10 days) timescales, have also been used in dendroclimatology. Using correlation coefficients between the mean temperature of pentads and tree-ring width indices, Vaganov et al. [17] reported the important interval of the tree growth season at four sites near the northern timberline. Naurzbaev and Vaganov [18] reconstructed early summer (June 17–July 11) temperature variations in east Taymir and Putoran over the last two millennia based on the response relationship between tree-ring width chronology and pentad climatic factors. Utilizing the weekly cambial activity of Scot pine, Seo et al. [19] analysed the radial growth variation in response to the weekly climate change during the growing season. Based on Chinese pine (*Pinus tabulaeformis*) ring-width chorology, Liu et al. [20] reconstructed February–early July precipitation for the Wu Dangzhao region and February–mid-July rainfall for the La Madong region, Inner Mongolia, China. However, all of the above studies were carried out within one timescale. It is necessary to explore the differences and similarities of the climate-growth relationships of trees at different timescales.

The Qinling Mountains form a significant geographic division line and serve as the most crucial boundary for climate and vegetation distribution in the middle of China due to their west-to-east alignment, which separates semi-arid areas from humid regions [21]. Many studies of past climate and environmental changes based on tree rings have been carried out in the Qinling Mountains and surrounding areas. In the western Qinling Mountains, Yang et al. [22] used a modified point-by-point regression method and multi-proxy indices to reconstruct the annual mean temperature change from 1500 to 1995 in west Qinling, and Chen et al. [23] reconstructed February–July mean temperature for Zhouqu back to AD 1650 based on Faxon fir ring-width standard chronology. In the central Qinling Mountains, Liu et al. [21] found that the climatic conditions of the previous year and the early summer temperatures had a pronounced influence on ring width on the southern and northern slopes, respectively. Based on the total tree-ring width and maximum latewood density, Garfin et al. [24] reconstructed February–April temperature for Foping and July–August precipitation for Xi’an. In the eastern Qinling Mountains, the ring widths of Chinese pine and high-elevation Huashan pine (*Pinus armandii*) in Funiushan were used to reconstruct the average maximum temperature of May–July and the winter half-year temperature [25, 26].

Dendroclimatic studies in Huashan along the northern margin of the eastern Qinling Mountains have provided some results [27–30]. Because there is a meteorological station with more than 60 years of observed data in Huashan, tree rings in this region can be used for more specific studies of the tree-ring response to climate.

In this paper, we utilized the tree-ring chronology of Huashan pine and its nearby meteorological data to (1) determine the differences and similarities of the tree growth response to climatic factors at daily, pentad, dekad and monthly timescales; (2) demonstrate that pentad is a more suitable scale for dendroclimatology; and (3) determine the limiting pentad climatic factor of tree radial growth in the Qinling Mountains. We believe that our study will be highly significant for the development of dendroclimatology and climatology.

## Materials and Methods

### Tree-ring data

**Fig 1:**
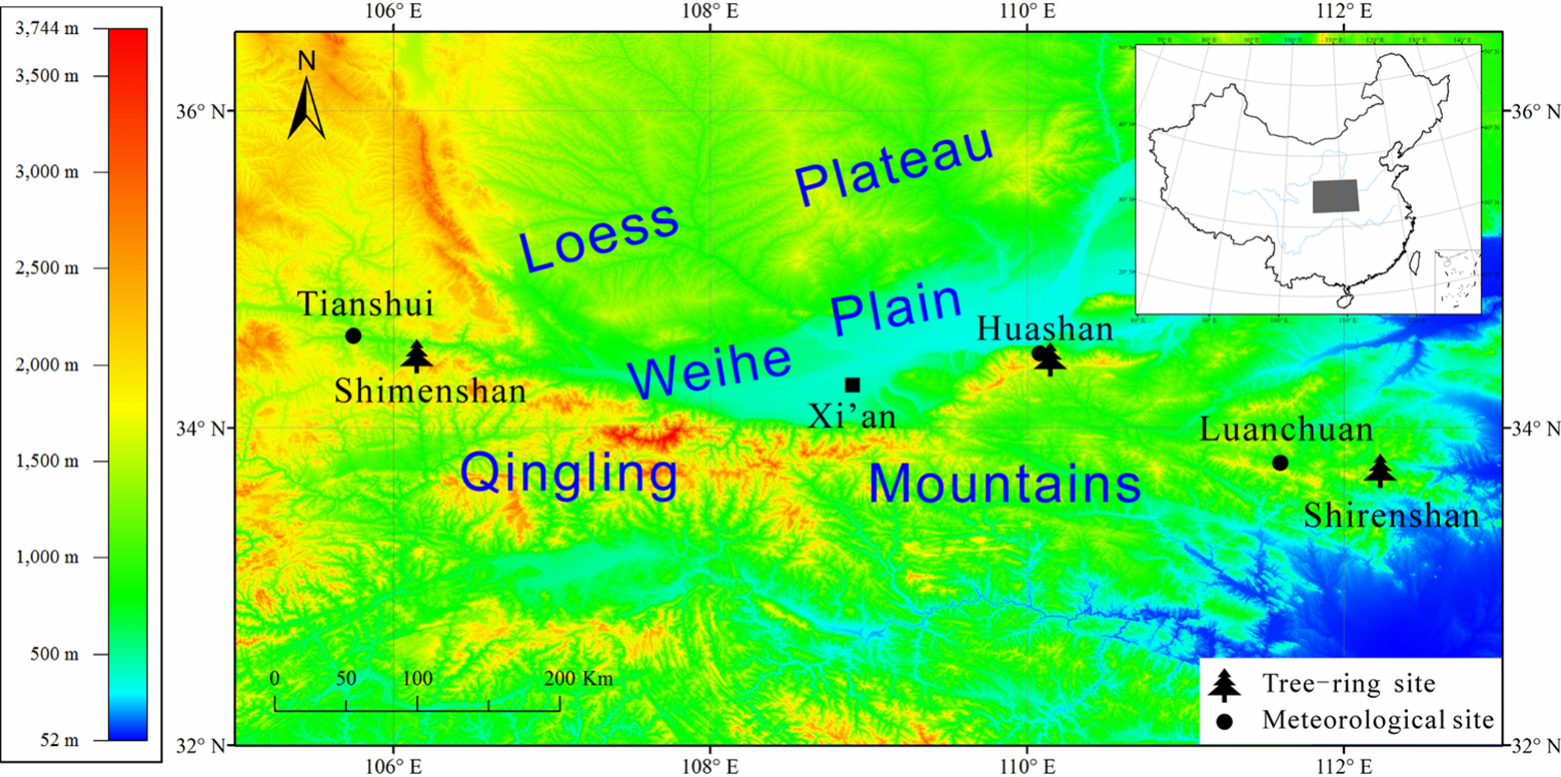
Location map of tree-ring sites and meteorological stations.

The three tree-ring chronologies used in this study were obtained from the National Centers for Environmental Information, NOAA (http://ncdc.noaa.gov/paleo/study). The sampling sites are located in the Qinling Mountains, north-central China (Fig 1). Huashan is located along the northern margin of eastern Qinling, and Shimenshan and Shirenshan are in western and eastern Qinling, respectively. The Shimenshan tree-ring data are Chinese pine earlywood density standard chronology [31], and the Shirenshan data are Chinese pine tree-ring width standard chronology [32]. The Huashan data are the originally measured tree-ring widths of Huashan pine at three sites [33]. As the three Huashan pine sampling sites were very close to each other and the ring-width series cross-dated very well [27], all of the increment cores were used to construct the tree-ring chronology using the ARSTAN program [34, 35]. Here, we used the standard chronology which preserves both low- and high-frequency information and mainly reflects the variations in climate.

### Climate data

The observed climate data of Huashan, Tianshui and Luanchuan were obtained from the China National Meteorological Center (http://ncc.cma.gov.cn/). These stations are the nearest meteorological stations to each sampling site (Table 1 and Fig 1). The annual mean temperatures of Huashan, Tianshui and Luanchuan are approximately 6.2°C, 11.1°C and 12.1°C, and the annual precipitation levels are 836 mm, 521 mm and 853 mm, respectively.

**Table 1:** Information on tree-ring sites and meteorological stations in the Qinling Mountains.

	Huashan	Huashan	Shimenshan	Tianshui	Shirenshan	Luanchuan
Longitude (°E)	110.08	110.08	106.15	105.75	112.23	111.60
Latitude (°N)	34.47	34.48	34.45	34.58	33.73	33.78
Altitude (m)	1950–2050	2065	2050–2100	1142	1675	750
Tree species	*P*. *armandii*		*P*. *tabulaeformis*		*P*. *tabulaeformis*	
Indices	Ring width		Earlywood destiny		Ring width	

### Methods

Huashan station has a similar altitude and climate environment to the sampling location of Huashan pine and thus reflects the trees’ living climate. Therefore, Huashan chronology and meteorological data were the major materials used to analyse the different responses of tree growth to climate at daily, pentad, dekad and monthly timescales. All of the climate data from the meteorological stations were processed using the same method. The climatic factors examined were precipitation and temperature (minimum, maximum and mean temperature).

**Fig 2:**
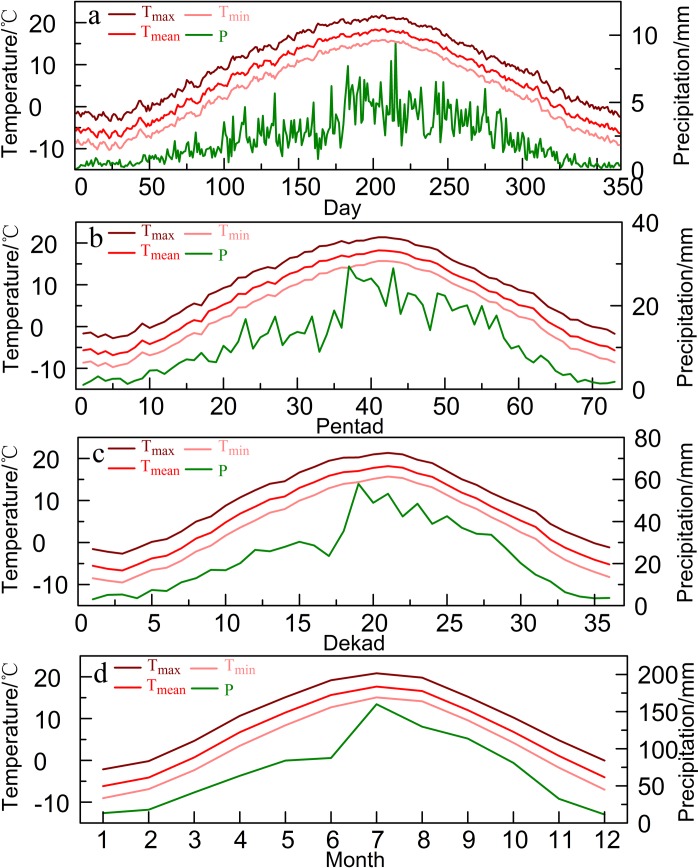
**Climatic factors of Huashan at daily (a), pentad (b), dekad (c) and monthly (d) timescales.** T_max_, T_mean_, T_min_ and P represent the maximum temperature, mean temperature, minimum temperature and total precipitation respectively.

The daily data were used to calculate the pentad, dekad and monthly values. For Huashan, we first generated the daily average of each climatic factor from 1953 to 2010 (Fig 2A). There is an extra day in a leap year: February 29th. The total precipitation of February 28 and 29 was used as the February 28 precipitation, and the average temperature of February 28 and 29 was used as the February 28 temperature. There are 73 pentads in a year. For example, the first pentad is January 1–5, and the 73rd pentad is December 27–31 [36]. The pentad precipitation is the total rainfall over a five-day period, and the temperature is the mean temperature of those five days (Fig 2B). There are three dekads in a month and 36 dekads in a year. The first dekad of a month extends from the first to the 10th day of the month; the following 10 days are the second dekad; and the 21st to the last day of the month is the third dekad. Similar to the pentad, the dekad precipitation is the sum of the daily rainfall, and the temperature is the mean value of the 10 days (Fig 2C).

At the daily, pentad, dekad and monthly timescales, a correlation function analysis method was used to identify the growth response of the trees to climate. A simple linear regression model was used to establish the reconstructed equation, and the split calibration-verification procedure was used to verify the reconstruction function [35, 37].

## Results and Discussion

### Tree response to climate at four timescales

The main growing season of Huashan pine is from approximately April to September [38, 39], and tree growth is affected by the climate of not only the current year but also the previous year [37]. Therefore, correlation analyses between ring widths and climatic variables were performed from October 1 of the previous year to September 30 of the current year at a daily timescale, from pentad 55 of the previous year to pentad 55 of the current year, from dekad 31 of the previous year to dekad 30 of the current year, and from October of the prior year to September of the current year at the pentad, dekad, and monthly timescales, respectively. According to the dendroclimatology method [35, 37], the response relationships of tree growth to climate were analysed during their common period from 1953 to 2005.

**Fig 3:**
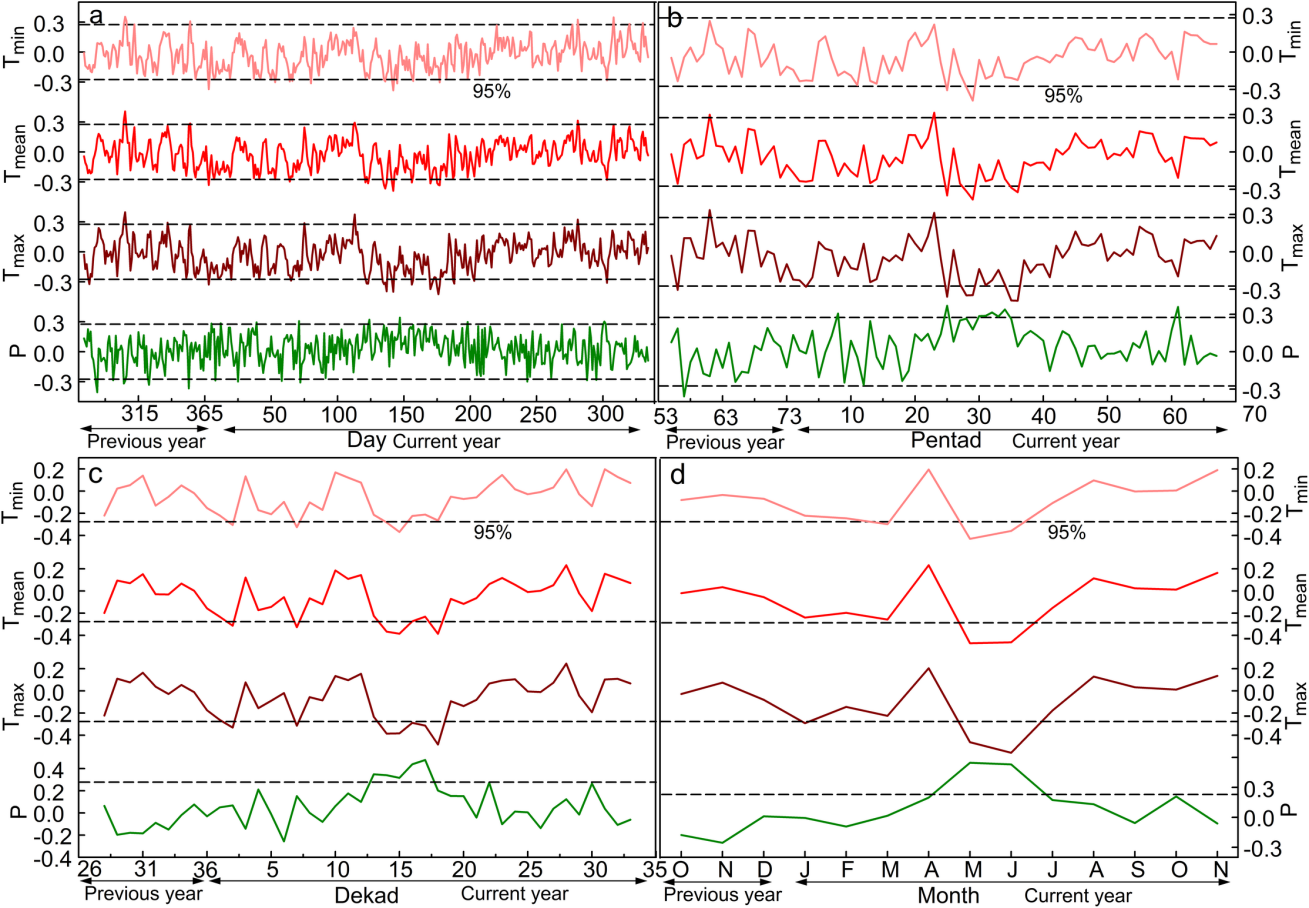
**Correlation coefficients between ring width and each climate element at daily (a), pentad (b), dekad (c) and monthly (d) timescales.** T_max_, T_mean_, T_min_ and P represent the maximum temperature, mean temperature, minimum temperature and total precipitation respectively.

Correlation analysis showed a remarkable relationship between tree growth and precipitation; this relationship was mainly positive, and a significant negative correlation only appeared at the daily and pentad timescales (Fig 3). The striking negative response relationship occurred during almost every period of a year at the daily timescale and only appeared at the end of the previous growing season at the pentad timescale. The high precipitation at the end of the previous growing season may saturate the soil, contributing to low aeration and low root growth [37]. Trees cannot store sufficient nutrients to utilize at the beginning of the next growing season, which does not result in the formation of a larger ring. Fritts [37] pointed out that, at certain times of one year, extremely high precipitation might influence environmental conditions which directly or indirectly limit tree growth. There was a remarkable correlation between chronology and temperature, which was mainly negative (Fig 3). Significant direct relationships were observed at the end of the previous growing season and the pre-growth period of the current season at the daily and pentad timescales. The higher temperature at the end of the previous season may promote the storage of carbohydrates, thus enhancing growth during the following year [37, 40]. Although cambial activity ceases at the end of the previous growing season, a higher temperature is beneficial for the trees and helps them to synthesize organic substances. These organic substances could be used to produce more wood in the next growing year [41]. A higher pre-growing season temperature may result in an earlier starting date of the growing season, thereby lengthening the growing season and leading to wider rings [42, 43]. These results indicate that the tree growth response to climate at the daily and pentad timescales can clearly reflect the influence of climate on trees to some extent.

In addition, we also calculated the correlation coefficients between tree-ring indices and all of the combinations of climate variables at each timescale to recognize the key climate factor influencing tree growth. There were 66,795 precipitation groups at the daily timescale, 2775 at the pentad timescale, 666 at the dekad timescale and 78 at the monthly timescale. Temperature (minimum, maximum and mean temperature) was divided into the same groups as precipitation. The maximum correlations between the tree-ring data and climate variables exhibited certain similar characteristics at the four timescales (Table 2). At all timescales, the tree-ring data had the strongest correlation with precipitation. Regardless of the precipitation and the three temperature factors, the absolute value of the maximum correlation coefficient increased as the timescales decreased. The numbers of days with the maximum correlations between the tree-ring data and the precipitation and temperature variables were similar for the four timescales, and the onset/end dates were approximate. The temperature values of the onset dates of the tree responses to precipitation and temperature were similar for the four timescales (Table 2 and Fig 2). This indicated that the temperature required for the highest speed of xylem cell production needed to reach suitable value [44, 45]. The above points indicate that the response of tree rings to climate at the four timescales is reasonably credible.

**Table 2:** Maximum correlations between tree-ring width and each climate element at different timescales.

Climate element	Time scale	Correlation coefficient	Onset Date	End Date	Number of days
Precipitation	Day	0.804	April 8	June 23	77(Days 98–174)
	Pentad	0.796	April 6	June 24	80(Pentads 20–35)
	Dekad	0.779	April 11	June 20	71(Dekads 11–17)
	Month	0.758	April 1	June 30	91(April–June)
Mean temperature	Day	-0.700	May 14	July 7	55(Days 134–188)
	Pentad	-0.687	May 16	June 29	45(Pentads 28–36)
	Dekad	-0.671	May 11	June 30	51(Dekads 14–18)
	Month	-0.625	May 1	June 30	61(May–June)
Minimum temperature	Day	-0.608	May 14	July 7	55(Days 134–188)
	Pentad	-0.589	May 16	June 29	45(Pentads 28–36)
	Dekad	-0.567	May 11	June 30	51(Dekads 14–18)
	Month	-0.549	May 1	June 30	61(May–June)
Maximum temperature	Day	-0.750	May 14	June 30	48(Days 134–181)
	Pentad	-0.739	May 16	June 29	45(Pentads 28–36)
	Dekad	-0.716	May 11	June 30	51(Dekads 14–18)
	Month	-0.655	May 1	June 30	61(May–June)

However, the differences in tree response to climate at different timescales are the emphasis of this study. The highest correlations were observed between the tree-ring data and all of the climate elements at the daily timescale. The superiority of daily timescale was found in dendroclimatic models. Dendroclimatic models with daily climate data were more efficient in identifying special climatic events that lasted only a few days but drastically influenced tree growth compared with dendroclimatic models using monthly climatic factors [46]. It was reported that the influences of temperature on the radial growth of *Quercus ilex* L. were more relevant at shorter time scales and explained the reason why the correlations between tree-ring width and precipitation were often more significant than those between ring width and temperature at monthly timescale in Western Mediterranean Basin [47]. The numbers of days and onset/end dates of the maximum correlations between the tree-ring data and each climate element were slightly different at the four timescales and were relatively precise at the daily scale. Thus, the daily timescale should be the best selection for research on tree growth and climate. However, the calculations at the daily timescale are too large; for example, there were 66,795 groups for each climate element in this study. In addition, the maximum correlation coefficients between the tree-ring data and climatic factors as well as their numbers of days and onset/end dates at the pentad scale were similar to those at the daily timescale, especially with respect to the first two important factors: precipitation and maximum temperature. This indicated that the pentad timescale could substitute the daily scale to a certain extent. In addition, the pentad timescale is also widely used in climate change [36, 48–50], agriculture [51] and tree physiology [17, 52] research. The response relationship of cambium activity in trees to climate was discovered by analysing the correlations between tree-ring structure parameter chronologies and pentad temperature in the Siberian Subarctic [52]. Therefore, comparatively speaking, the pentad timescale is better for studying the relationships between climate change and tree radial growth in Qinling Mountains.

The Twenty-four Solar Terms, Chinese traditional terms, precisely describe the climate change characteristics in the seasonal cycle. They present some meaningful directions for seasonal sequence, phenology change and crop growth, especially for the agriculture and forestry production [53]. The highest response of radial growth of Huashan pine to precipitation at pentad timescale (Table 2) happened to be the season from Pure Brightness to the Summer Solstice [53]. During this seasonal interval, precipitation of Huashan remained in a relatively stable level (Fig 2C). When Pure Brightness arrives, trees and grasses begin to bud and everything begins to grow. The Summer Solstice indicates that Northern China enters into midsummer and everything is in the most robust growth time in a year [54]. The seasonal time of the highest pentad temperature response to tree growth was from 10 days after the Beginning of Summer to 5 days after the Summer Solstice. The Beginning of Summer indicates that summer arrives in Northern China and trees and crops turn into the exuberant growth time [54]. Since 10 days after the Beginning of Summer, mean pentad temperature was larger than 11°C (Fig 2C), which made Huashan pine growth be subject to temperature in stress [15]. And we also found that the onset date of pentad 20 was close to the beginning of the trees’ growing season which was from the observed phenological data at Xi’an, the nearest phenological station to Huashan [49, 55].

### Advantages of the pentad timescale compared to the monthly timescale

Monthly climate is not the best choice for the studies on relationships between tree growth and climate change at sometimes. Through collecting micro-cores from five even-aged Chinese pine trees at weekly intervals, Liang et al. [44] discovered cell division in the cambial zone of trees in North China started within the third week of May rather than at the beginning or the end of a month. It was also found that climate had a more accurate response relationship with tree growth at a timescale shorter than one month in the Inner Mongolia and the April 1–July 10 precipitation was reconstructed [56]. The response of tree growth to climate is stronger and more precise at the pentad timescale compared with the monthly timescale in this study (Table 2). The absolute values of the maximum correlation coefficients increased by 0.038 for precipitation, 0.040 for minimum temperature, 0.062 for mean temperature and 0.084 for maximum temperature from the pentad to the monthly timescale. To further explain the superiority of the pentad timescale, we determined the maximum correlation between the tree-ring data and the precipitation for all 18 pentad groups. Although 18 pentads had the same number of days with a 3-month period, their coefficients and dates of maximum correlation were different (Table 3). The maximum coefficient for the 18 pentads (0.766) was higher than that (0.758) for the 3-month period. There were also differences in the maximum correlation between the tree-ring data and temperature for 12 pentads and 2 months. The correlation coefficients increased from 0.018 to 0.029 (Table 3).

**Table 3:** Maximum correlations between the tree-ring data and climate factors at pentad and monthly timescales with the same number of days.

Climate element	Time scale	Correlation coefficient	Onset Date	End Date	Number of days
Precipitation	Pentad	0.766	March 27	June 24	90
	Month	0.758	April 1	June 30	91
Mean temperature	Pentad	-0.654	May 11	July 9	60
	Month	-0.625	May 1	June 30	61
Minimum temperature	Pentad	-0.567	May 11	July 9	60
	Month	-0.549	May 1	June 30	61
Maximum temperature	Pentad	-0.683	May 11	July 9	60
	Month	-0.655	May 1	June 30	61

High-quality past climate reconstruction depends on successful calibrations and quantitative relationships between tree-ring indices and instrumental data of climatic factors [57, 58]. According to the principle of dendroclimatology, we established reconstructed functions (omitted in this paper) for precipitation of pentads 20–35 and precipitation from April–June. The functions were examined using a split calibration-verification procedure [35]. Due to its superior test parameters, the reconstruction function of the precipitation of pentads 20–35 was more reliable and stable (Table 4). For example, the correlation coefficient (r), reduction of error test (RE) and coefficient of efficiency (CE) increased, and the sign test (ST) was more significant [35, 37]. These results further indicated that the pentad timescale was superior for dendroclimatic research.

**Table 4:** Statistics of the split calibration-verification model for monthly and pentad precipitation reconstruction.

Time scale	Calibration				Verification					
	Period	r	ST	t	Period	r	RE	CE	ST	t
Pentad (20–35)	1953–1979	0.772	21**	4.884	1980–2005	0.821	0.653	0.592	19*	5.154
	1979–2005	0.820	21**	5.503	1953–1978	0.756	0.619	0.504	23**	4.232
	1953–2005	0.796	44**	7.255						
Month (April–June)	1953–1979	0.751	21**	4.765	1980–2005	0.769	0.601	0.523	18	5.329
	1979–2005	0.775	20*	5.776	1953–1978	0.731	0.587	0.468	22**	4.150
	1953–2005	0.758	43**	7.165						

*Significant at the 0.05 level

**Significant at the 0.01 level

r, correlation coefficient; RE, reduction of error test; CE, coefficient of efficiency; ST, sign test; t, the product means test.

Tree radial growth had a good response to temperature and precipitation at different timescales, but the highest response happened to the relationship between the tree-ring width of Huashan pine and the precipitation of pentads 20–35. Therefore, according to the principle of dendroclimatology [35, 37], the precipitation from pentad 20 to pentad 35 is the major determinant of Huashan pine growth, which can be explained with explicit physiology. Monsoon rainfall plays an important role in the climate change and vegetation condition in northwest China and the pentad precipitation represents the variation of monsoon rains [50]. From pentad 20, the mean temperature is higher than 5°C, above which Huashan pine enters a rapid growth period [39]. Once the temperature reaches a specific suitable value and tree radial growth starts, factors other than temperature generally become limiting [37,59]. Higher rainfall is better for cambial activity and is conducive to the generation of additional and larger cells, which benefits wider ring formation [27, 28]. With the coming of the rainy season, precipitation increases. From pentad 36, high amounts of rainfall are sufficient to replenish soil moisture and meet the demands of tree growth. The role of precipitation in tree radial growth weakens [37]. This tree growth pattern controlled by precipitation during the growing season was widely found in northwest China, such as the Xiaolong Mountain [60], the north-western Qilian Mountain [61] and the north Helan Mountain [62].

### Application of the pentad timescale in western and eastern Qinling

Chen and Yuan [31] indicated that the maximum temperature from May to June was the major climate factor controlling the mean earlywood density (EWD) of Chinese pine trees in Shimenshan in western Qinling. Based on the EWD chronology [31], we analysed the response of tree growth to maximum temperature at the pentad and monthly timescales. The chronology had the strongest correlation with the maximum temperature of pentads 28–32 (May 16–June 9). The coefficient was higher than that between chronology and May–June maximum temperature (Table 5), demonstrating that the major factor affecting the mean earlywood density was the maximum temperature of pentads 28–32, in addition to that from May to June. In eastern Qinling, Shi et al. [32] found that the maximum temperature from May to June was also the main factor limiting the ring width of Chinese pine trees in Shirenshan. Similarly, the maximum temperature of pentads 28–33 (May 16–June 14) was the main limiting factor. The absolute value of the correlation coefficient increased by more than 0.1, demonstrating that the pentad was indeed a better timescale for tree-climate response. However, we should be conscious that these results merely show a deeper understanding of the response relationship between tree-ring indices and climatic factors at different timescales in the Qinling Mountains.

**Table 5:** Comparison of the pentad and monthly timescales as used in the Qinling Mountains.

Sites	Huashan	Shimenshan	Shirenshan
Period of time	1953–2005	1953–2008	1957–2007
Climate element	Precipitation	Maximum temperature	Maximum temperature
Highest correlation (Month)	r = 0.758, April–June	r = 0.638, May–June	r = -0.442, May–June
Highest correlation (Pentad)	r = 0.796, April 6–June 24	r = 0.697, May 16–June 9	r = -0.543, May 16–June 14

## Conclusions

By utilizing the tree-ring width chronology of Huashan pine from Huashan, north-central China, we found that the response relationship between tree radial growth and climate varied at the daily, pentad, dekad and monthly timescales. As the timescale decreased, the maximum correlations between the tree-ring data and climatic factors increased. The response of tree-growth to climate was stronger and more precise at short timescales. Compared to the other three timescales, the pentad was more suitable for analysing the response of tree growth to climate. When the pentad climate was used to analyse the climate-growth response in the Qinling Mountains, the major climatic factor limiting the growth of Huashan pine from Huashan was the precipitation of pentads 20–35 (from April 6 to June 24) rather than the well-known April–June precipitation. The mean earlywood density of Chinese pine trees in Shimenshan was mainly limited by the maximum temperature of pentads 28–32 (May 16–June 9) and not by the May–June maximum temperature. The major factor limiting the ring-width of Chinese pine trees in Shirenshan was the maximum temperature of pentads 28–33 (May 16–June 14) rather than that of May–June.

In general, because of different physiological properties and living environments, a climate signal sometimes exists in the tree ring that is likely not monthly climate but rather pentad climate. If the response of trees to monthly climate fails to meet the requirements of reconstruction, the pentad climate is a better selection criterion.
